# Successful Management of Refractory Headache and Facial Pain due to Cavernous Sinus Meningioma with Sphenopalatine Ganglion Radiofrequency

**DOI:** 10.1155/2014/923516

**Published:** 2014-09-29

**Authors:** Foad Elahi, Kwo Wei David Ho

**Affiliations:** Center of Pain Medicine, University of Iowa, Iowa City, IA 52242, USA

## Abstract

Headaches and facial pain can be extremely difficult to manage for the patient and the clinician. In the medical literature, it has been suggested that the autonomic reflex plays an important role in the pathophysiology of facial neuralgia. The sphenopalatine ganglion is the largest parasympathetic ganglion outside the cranium. It is an easy accessible target for pain management. The application of radiofrequency nerve ablation was described in the medical literature. In this case report, we describe a 54-year-old female. She was diagnosed with a cavernous sinus meningioma. She underwent surgical resection and gamma knife radiosurgery. She was suffering from an intractable hemifacial pain for many years. Her pain started shortly after surgery and continued throughout many years. Sphenopalatine ganglion block in multiple occasions was able to provide temporary relief. The patient's intractable hemicranial headaches and hemifacial pain responded to the sphenopalatine ganglion radiofrequency nerve ablation. The pain response remained unchanged for 12 months after procedure. This case report increased our current knowledge about the sphenopalatine ganglion role in the headache and facial intractable pain management. The failure of available antalgic medications to adequately control pain in similar patients underscores the need to develop an algorithm for therapies.

## 1. Introduction

Cavernous sinus meningiomas occur in 0.5 per 100,000 persons in the general population. Cavernous sinus meningiomas can present with facial pain or headaches. Due to the anatomical location, a cavernous sinus meningioma is a difficult meningioma to treat. The surgical resection of cavernous sinus meningiomas usually results in severe neurological deficits. Irrespective of the recent advances in microsurgery, stereotactic radiosurgery, and fractionated radiotherapy, facial pain and headaches can be a long-term devastating problem that may persist after successful tumor treatment. The cavernous sinus has a compact space containing multiple important structures, including the cerebral artery, ocular motor nerves, optic nerves, and pituitary body. Despite technical advances regarding microsurgical resections of cavernous sinus meningiomas, they are rarely completely resected. After partial or subtotal tumor removal, the probability of recurrence remains significant, and the possibility that patients remain symptomatic is high [1]. Due to compression on cranial nerves, symptoms of cavernous sinus meningiomas often include facial pain and headaches.

Pharmacological treatment of these painful conditions is not always successful. Therefore, the facial neuralgia and headaches are a significant challenge in management.

It has been suggested that the autonomic reflex plays an important role in the pathophysiology of headaches and facial neuralgia. The key structure in the expression of cranial autonomic symptoms is the sphenopalatine ganglion (SPG), also known as the pterygopalatine ganglion. Parasympathetic fibres from the SPG innervate facial structures, cerebral meninges, and meningeal blood vessels. The SPG is the largest group of neurons outside of the cranial cavity. It contains sensory (maxillary nerve), parasympathetic (greater petrosal nerve), and sympathetic (superior cervical ganglion) nervous systems [2].

The spehnopalatine ganglion is a great target for pain physicians, neurologists, and surgeons. Neural blockade or electrical stimulation of the SPG has been used to treat multiple types of headaches and facial neuralgias. In patients with either episodic or chronic cluster headaches, radiofrequency ablation of SPG improved pain symptoms [3]. Short-term electrical stimulation of the SPG also appeared effective for episodic cluster headaches [4]. Endoscopic SPG blockade by a mixture of anesthetics and corticosteroids was similarly shown to be useful in cluster headaches refractory to medications [5]. In migraine, nasal lidocaine-induced SPG block and electrical stimulation provided significant relief. In posttraumatic headache, radiofrequency ablation of SPG similarly results in long-term relief [6–8]. Blockade of SPG has also been reported to be successful in treating facial neuralgia. In sphenopalatine neuralgia, stereotactic radiosurgery has been used as a successful treatment. Trigeminal neuralgia was also satisfactorily treated by nerve block of SPG. A case series showed that atypical facial and head pain can be treated with pulsed radiofrequency of SPG [9, 10].

Here, we report a case of refractory headache and facial neuralgia induced by a cavernous sinus meningioma. It was successfully treated with radiofrequency ablation of the SPG.

## 2. Case

**Figure 1:**
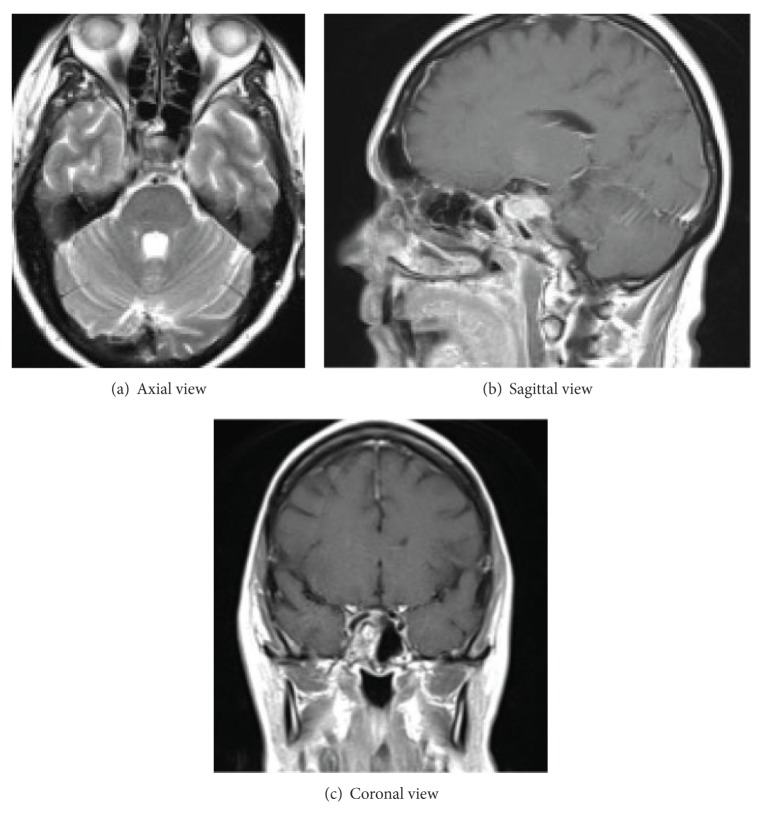
MRI: axial (a), sagittal (b), and coronal (c) views show right cavernous sinus tumor remnant after surgery and radiation.

The patient is a 54-year-old female and she was diagnosed with a cavernous sinus meningioma after symptoms of hemifacial pain for two years. Tumor was 3 centimeters by 2 centimeters in diameter on her initial MRI. She underwent a partial resection of the cavernous sinus meningioma, and the remnant was subsequently treated by gamma knife radiosurgery (see Figure 1).

The patient had a greater than seven-year history of constant right-sided headache along with right facial, retroorbital, and upper teeth pain after tumor resection. When she was referred to the pain clinic, she rated her pain 7/10 on the visual analog scale on daily basis. Her pain ranged from 7 to 10, occasionally accompanied with nausea and rarely with vomiting. She denied double vision, photophobia, and aura. Pain is persistent with no identifiable aggravating or alleviating factor.

**Figure 2:**
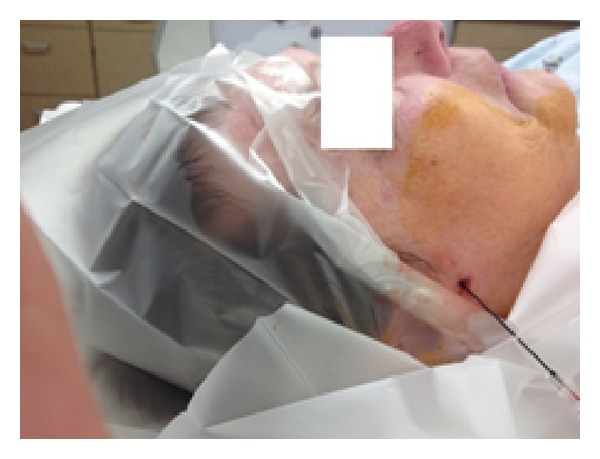
Location of needle insertion.

**Figure 3:**
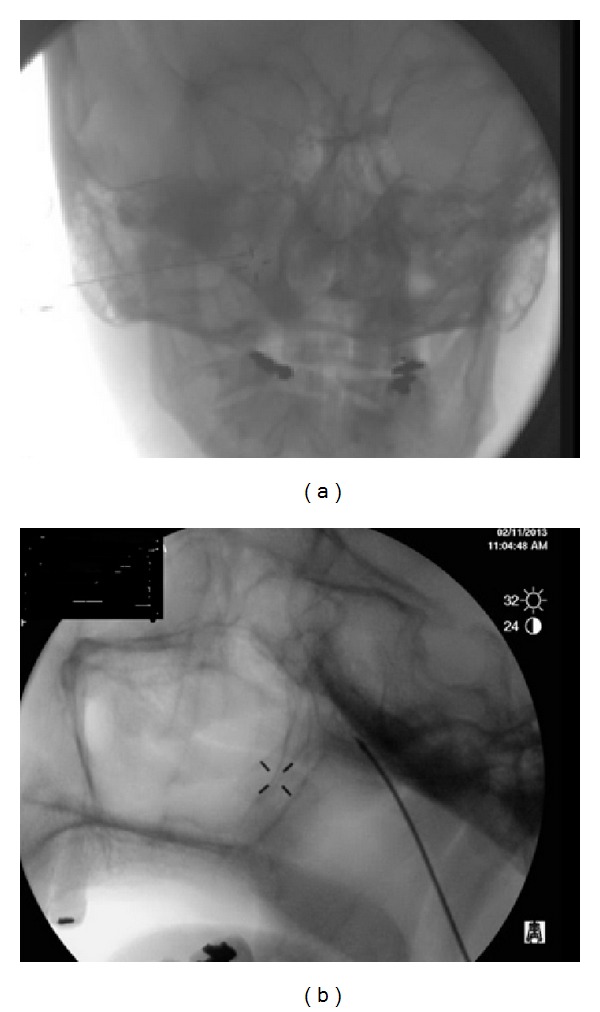
Anteroposterior view X-ray (a) and lateral X-ray (b) show the final radiofrequency needle position at the sphenopalatine fossa.

She was seen by her neurosurgeon, neurologist, radiation oncologist, and endocrinologist. Among laboratory and imaging, she had normal hormonal workup and electroencephalography. Multiple imaging reported stable tumor size and no evidence of tumor progression for five subsequent years (Figures 1, 2, and 3).

All physical examinations including cranial nerves were normal. She experienced multiple combinations of medications including carbamazepine, gabapentin, topiramate, tramadol, hydrocodone, hydromorphone, and many other combinations with no significant relief with monotherapy or combination of medications.

Based on her presentation, she was scheduled for sphenopalatine ganglion block under fluoroscopy guidance. The SPG blocks in multiple occasions provided short-course pain relief and in two occasions successfully aborted pain exacerbation. Based on this observation, we decided to proceed with radiofrequency nerve ablation in order to provide a long-term pain relief.

### 2.1. SPG-Radiofrequency Nerve Ablation (RFA) Procedure

The patient was placed supine on the fluoroscopy table. The pterygomaxillary fissure was localized using lateral fluoroscopy view. Skin and underlying tissue were anesthetized with lidocaine 1%. A 100-millimeter long, 21-gauge radiopaque radiofrequency needle with 5-millimeter active tip (BVM Medical Limited, Leicestershire, UK) was inserted inferior to the zygomatic arch in the direction of the sphenopalatine foramen. Final location of the needle tip was verified under fluoroscopy with anterior/posterior and lateral views.

At this stage, the radiofrequency needle tip was stimulated with 50 Hertz. The stimulation resulted in paresthesia in the nose and the skin in between nasolabial midline region. We repeated the sensory stimulation a few times to make sure that the paresthesia does not distribute in the maxillary nerve distribution. A repeat X-ray on anterior/posterior plane helps to make sure that the needle tip did not move during the stimulation. After obtaining enough confidence about the physiological response and radiological verification of a correct positioning, 0.5 cc of lidocaine 2% was injected and radiofrequency cannula was connected and RFA of lesion was performed at 80 degrees of centigrade with a duration of 90 seconds. RF cannula was removed after one minute to permit the cannula to go through the cooling process in order to prevent vascular damage.

After the SPG-RFA, she reported great pain relief. She reported pain score of 2-3 on daily basis during her followup. She was able to wean off narcotic medications completely. Her current total daily medications are gabapentin 900 mg and amitriptyline 25 mg. She is able to manage occasional exacerbation of pain with 400 mg of ibuprofen. At the 12-month followup, she expresses her satisfaction with pain relief, and the SPG-RFA benefit remains the same.

## 3. Discussion

Cavernous sinus meningioma has been a difficult meningioma to treat because of its adjacent structures. It often causes symptoms including facial neuralgia and headaches because of its proximity to cranial nerves.

Despite the continuous development of new and refined medical approaches as well as surgical techniques over the recent decades, the management of headaches and facial pain still remains a challenge for neurosurgeon and radiation oncologists.

The SPG is the largest and most superior ganglion of the sensory, sympathetic, and parasympathetic nervous systems.

Blockade of the SPG has been demonstrated to be useful in the management of multiple pain syndromes of the head and face. It has been applied successfully to cluster headaches, migraine, posttraumatic headache, trigeminal neuralgia, sphenopalatine neuralgia, and atypical facial and head pain.

Approach to the SPG is fairly safe, and, in the medical literature, there is a versatile technique with multiple variations including nerve block, radiofrequency, and electrical stimulation. However, most reports still remain at the level of observational studies or case series. More specific case-controlled trials are necessary to solidify these findings and further characterize the indications for this intervention.

Here, we report a case of chronic refractory hemifacial neuralgia and headache status after surgery and radiation of cavernous sinus meningioma. The neuralgia and headache were successfully treated with radiofrequency ablation of the SPG, and the patient reported satisfactory pain relief.

The successful treatment demonstrated in this case report gives further support for the SPG blockade and radiofrequency as a useful technique in treating headaches and facial neuralgia.

One of the most important side effects of SPG-RFA is localized loss of sensation in distribution of the maxillary nerve due to the anatomical arrangement of the maxillary nerve in the pterygopalatine ganglion. Proper electrophysiological testing is the key element to prevent this complication.

## 4. Conclusion

There is no treatment guideline for similar patients in the literature. We may look forward to a clinical trial in the future, but the paucity of cases makes that a dilemma. This case report will be a great addition to the medical literature for future references if pain management remains the main goal for the surgeons, who are treating tumor involvement in the cavernous region.
